# Gene mining of immune microenvironment in hepatocellular carcinoma

**DOI:** 10.1097/MD.0000000000030453

**Published:** 2022-09-02

**Authors:** Zhi-Wei Xu

**Affiliations:** Department of Hepatobiliary Surgery, the First Affiliated Hospital of Guangxi Medical University, Guangxi Medical University, Nanning, China.

**Keywords:** bioinformatic analysis, hepatocellular carcinoma, immune microenvironment

## Abstract

Hepatocellular carcinoma (HCC) is a common malignant tumor worldwide with a poor prognosis. Recent studies have shown that the occurrence, development and prognosis of liver cancer are closely related to tumor microenvironment (TME) and tumor immune infiltration.

**Methods:**

Therefore, important information on various diseases can be obtained from public databases such as The Cancer Gene Atlas (TCGA), and ideas or schemes that may be effective for the treatment of various diseases can be screened and analyzed by screening various conditions. In this study, 424 cases of liver hepatocellular carcinoma (LIHC) in the TCGA database and CIBERSORT algorithm were used to calculate the proportion of tumor-invasive immune cells. Combined with the clinical data from TCGA database, it was concluded that T cells regulatory (Tregs) were correlated with the development and prognosis of HCC. Cox regression analysis was used to screen differentially expressed genes, and survival analysis was performed according to the screened differentially expressed genes to see whether there was a significant association with the prognosis of HCC. Then gene ontology and kyoto encyclopedia of genes and genomes analysis of differentially expressed genes were carried out to explore the possibility of differentially expressed genes becoming potential therapeutic targets of HCC.

**Results:**

Finally, I identified the gene centromere protein o (CENPO), which is associated with immune cells and improve the prognosis of HCC.

**Conclusion:**

CENPO may be a potential biological therapeutic target for hepatocellular treatment.

## 1. Introduction

The incidence and mortality of liver cancer are increasing year by year in the world, and liver cancer has become the second highest cause of cancer mortality in the world.^[1]^ At present, hepatocellular carcinoma (HCC) accounts for 90% of liver cancer. Nonalcoholic steatohepatitis is gradually becoming the most common risk factor leading to HCC, and nonalcoholic steatohepatitis has a specific and unique pathogenesis, so I need to find new targeted therapy or immunotherapy to improve the prognosis of liver cancer and HCC.^[2]^

Tumor microenvironment (TME) is the microenvironment where the tumor is located. It not only contains tumor cells, but also a variety of cells, such as epithelial cells, fibroblasts, vascular endothelial cells, and a large number of immune cells with different functions.^[3]^ Many studies have shown that the change of TME can normalize tumor cells, re-culture stromal cells and play an anti-tumor role.^[4]^ At the same time, innate immune cells and adaptive immune cells can interact with tumor cells, change the progress of tumor cells and improve the occurrence, development and prognosis of tumor,^[5]^ for example, studies have shown that Kupffer cells can enhance the response of immune T cells, regulate the growth of tumor cells together with macrophages, and inhibit the development of tumor.^[6]^ In recent years, it has been reported that the prognosis of HCC is related to immune cells, and the types and expression of immune cells in HCC caused by different etiologies are different.^[7]^ The staging of liver cancer is closely related to its prognosis, which needs scientific staging guidance and treatment plan.^[8]^ Due to the limited treatment options for advanced HCC, many patients are difficult to adapt to the treatment methods of hepatectomy and liver transplantation. Drug immunotherapy is particularly important.^[9]^ Immunotherapy for HCC has gradually attracted clinical attention and application.^[10]^

Therefore, this study attempts to use the liver hepatocellular carcinoma (LIHC) data of The Cancer Gene Atlas (TCGA) database to explore and study whether there is correlation between immune cells in different stages of HCC and whether there are differences in immune cells in different stages of HCC, and find out the possible target genes. According to the literature reports, convulxin will induce the release of IL-10 and the change of the number of T cells,^[11]^ and IL-10 will combine with IL-10 receptor to activate janus tyrosine kinase-signal transducer and activator of Tranion signal pathway.^[12]^ At the same time, it is also hoped that more antitumor drugs can be developed, such as cucurbitacin B, which is considered to have significant anti-tumor effects and is related to janus tyrosine kinase-signal transducer and activator of Tranion signal pathway and mainly acts on gastric cancer.^[13]^ In addition to cucurbitacin B, the inhibitory effect of chalcones on glioblastoma,^[14]^ 1,2,4,5-tetraoxanes analogues on malaria,^[15]^ and the inhibitory effect of phytol on CYP450^[16]^ also need our attention, which is conducive to our future selection and treatment of targeted therapeutic drugs for liver cancer.

## 2. Materials and Methods

### 2.1. Data collection and processing

The TCGA database is a very large genomics database with multiple cancer types which has genomics data of various cancers and genomics data of normal samples matched with corresponding cancers (https://portal.gdc.cancer.gov/).^[17]^ In this study, gene expression ribonucleic acid (RNA)-seq data (n = 424), clinical information (n = 469), and survival data (n = 463) were gained from TCGA database with level 3, which was downloaded through the Xena the University of California Santa Cruz Xena (http://xena.ucsc.edu/).^[18]^ CIBERSORT algorithm^[19]^ was used to calculate the transcriptome RNA seq data, and the number of repetitions was 1000. I extracted highly significant data with *P* < .05, matched them with clinical data, grouped them according to the situation of stage, and then drew the landscape histogram of immune cells in different stages through the program package ggplot2 (version 3.3.5) of R software (version 4.1.2, https://cran.r-project.org/bin/windows/base/old/4.1.2/). The statistical analysis of this part is carried out by R software.

### 2.2. Screening of differential genes

I divided RNA SEQ data into tumor data and normal data, and calculated and screened them through deseq2 package^[20]^ (version 1.34.0) of R software. The screening conditions were log2 fold change >1 and padj <0.05. In this way, the screened differential genes can be considered to be highly significant and differential. The statistical analysis of this part is carried out by R software. Subsequently, I tested whether the genes I screened were different through 2 data (GSE25097, GSE36376) in the Gene Expression Omnibus (GEO, http://www.ncbi.nlm.nih.gov/geo/)^[21]^ database. The statistical analysis of this part is carried out by GraphPad Prism (version 8.0.2, https://www.graphpad.com/).

### 2.3. Survival analysis

I use log rank test to calculate the survival curve of the screened differential genes related to TME. The platform is R software, the program package is survival (version 3.2-13), and the set threshold is *P* < .05, so I can think that the differential genes have differences in the survival and prognosis of HCC. Survival data (n = 363) is the survival data after screening and removing the paracancerous data. The pictures of Kaplan–Meier survival analysis are also produced by R software. The statistical analysis of this part is carried out by R software.

### 2.4. Cox regression analysis

I established a univariate Cox regression model to analyze the survival data, calculated the risk ratio of the screened differential genes, and further screened the top gene on this basis. The threshold of the top gene was set as pcutoff <0.05, and the 95% confidence interval of the risk ratio was one side of 1, that is, betIen 0 to 1 or >1. After selecting the top gene, I can make the forest map of gene risk ratio according to the calculated data. The platform is R and the program package is forestplot (version 2.0.1). The statistical analysis of this part is carried out by R software.

### 2.5. Heatmaps

The correlation heat map and clustering heat map of immune cells are also drawn by R software, and the program packages are corrplot (version 0.92) and heatmap (version 1.0.12), respectively. The correlation heat map of immune cells is calculated by Spearman correlation analysis algorithm.

### 2.6. Gene ontology and KEGG enrichment analysis

I will conduct go, kyoto encyclopedia of genes and genomes (KEGG) enrichment analysis on the finally screened genes. The platform is R and the program package is org Hs. e.g..db (version 3.14.0), clusterprofiler^[22]^ (version 4.2.2), enrichplot (version 1.14.2), and ggplot2. Go analysis reveals the main functions of genes in biological processes, cell components and molecular functions. KEGG analysis mainly analyzes the enrichment pathway of genes. I divided the expression of centromere protein o (CENPO) into high expression and low expression. After calculation, *P*-value < .05 was used as the screening threshold. The statistical analysis of this part is carried out by R software.

### 2.7. Gene set enrichment analysis

GSEA uses a predefined gene set to sort the genes according to the degree of differential expression, and then tests whether the gene set is enriched at the top or bottom of the sorting table. I will conduct GSEA on the finally screened genes. The platform is R and the program package is org Hs. e.g..db, clusterprofiler and enrichplot. I use C5 gene sets v7 0 collections were calculated as the target gene set, with *P* value <.05 and *q*-value <0.05 as significant. The statistical analysis of this part is carried out by R software.

### 2.8. Violin plot and box plot

The comparison of 22 immune cells shown in the violin diagram is calculated by Wilcoxon rank sum algorithm. The comparison of gene expression is divided into low expression and high expression based on the median of gene expression. It is also calculated by Wilcoxon rank sum algorithm. The above drawing and calculation are carried out on the R software platform, and the program package is vioplot (version 0.3.7). Box plot is also calculated and drawn by Wilcoxon rank sum algorithm on R software.

### 2.9. PPI network construction

The protein protein interaction (PPI) network of the screened genes was obtained through the search tool for the retrieval of interacting genes (https://string-db.org/)^[23]^ and imported into Cytoscape software^[24]^ (version 3.9.1) for reconstruction.

### 2.10. Scatter diagram

The scatter diagram is drawn on the platform of GraphPad Prism, and the statistical method is nonparametric *t* test.

## 3. Results

### 3.1. Data and immune cell expression estimation

**Figure 1:**
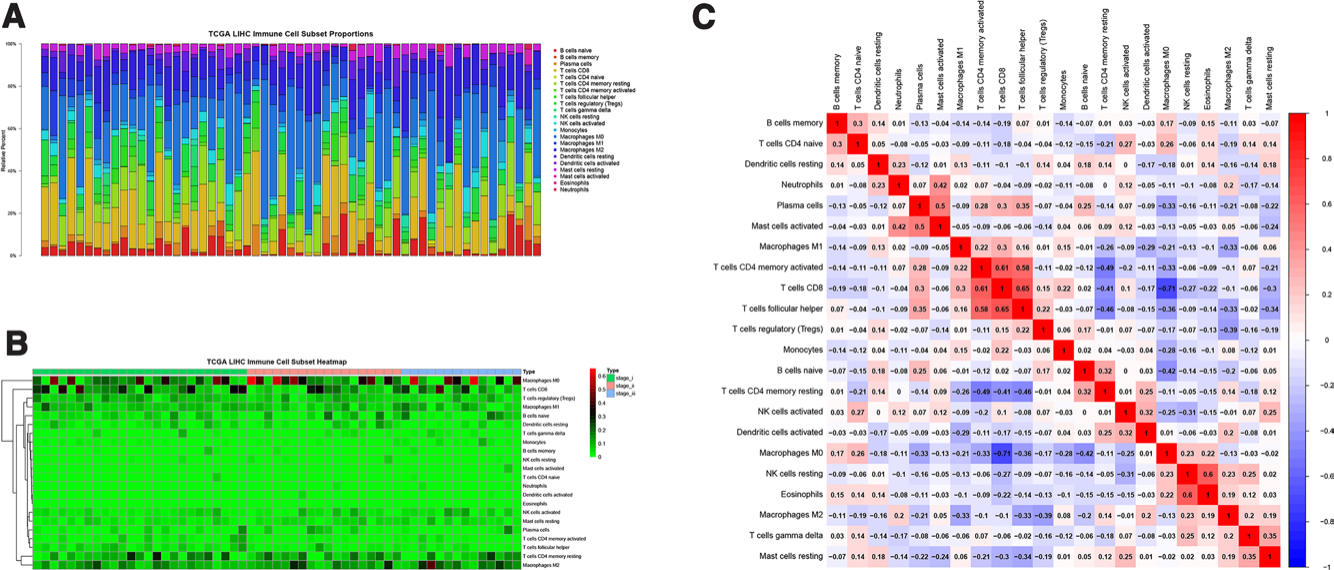
(A) The immune cell landscape histogram of patients with *P* < .05 was screened by ciberport algorithm. (B) Cluster Heatmap of 22 immune cells. (C) The correlation analysis of 22 immune cells was performed by Spearman.

In this study, I calculated the gene expression RNA sequence data (n = 424) of LIHC data in TCGA database in Xena the University of California Santa Cruz Xena with CIBERSORT algorithm, the number of repetitions was 1000, and then extracted the data with high significance, that is, *P*-value <.05. In this way, I roughly estimated the approximate expression data and proportion of 22 immune cells in each patient (Fig. 1A). Then I matched and grouped them by the stage according to the clinical data (n = 469). Finally, it was divided into stage I, stage II, and stage III.

### 3.2. Further analysis of immune cells

**Figure 2:**
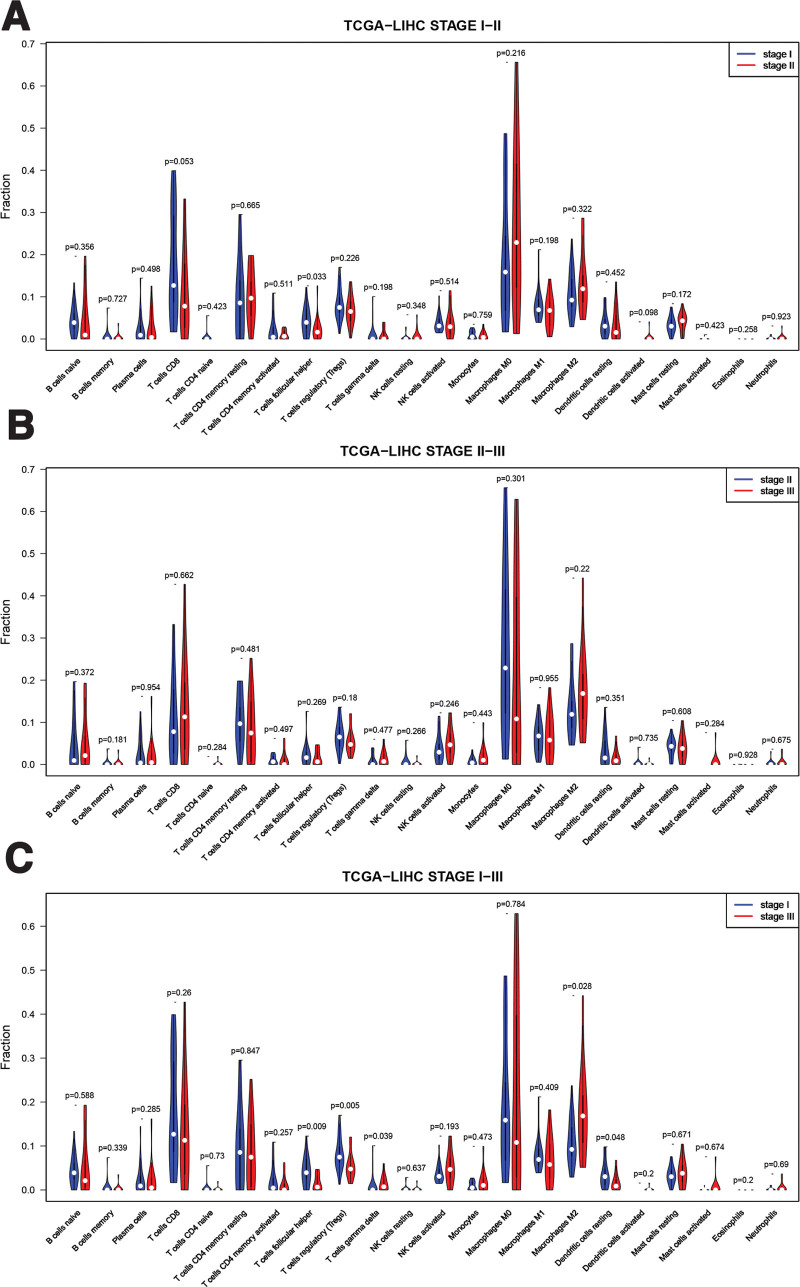
(A) The contents of stage I and stage II of immune cells in patients in TCGA database were calculated by Wilcoxon rank sum. (B) The contents of stage II and stage III of immune cells in patients in TCGA database were calculated by Wilcoxon rank sum. (C) The contents of stage I and stage III of immune cells in patients in TCGA database were calculated by Wilcoxon rank sum. TCGA = The Cancer Gene Atlas.

**Figure 3.**
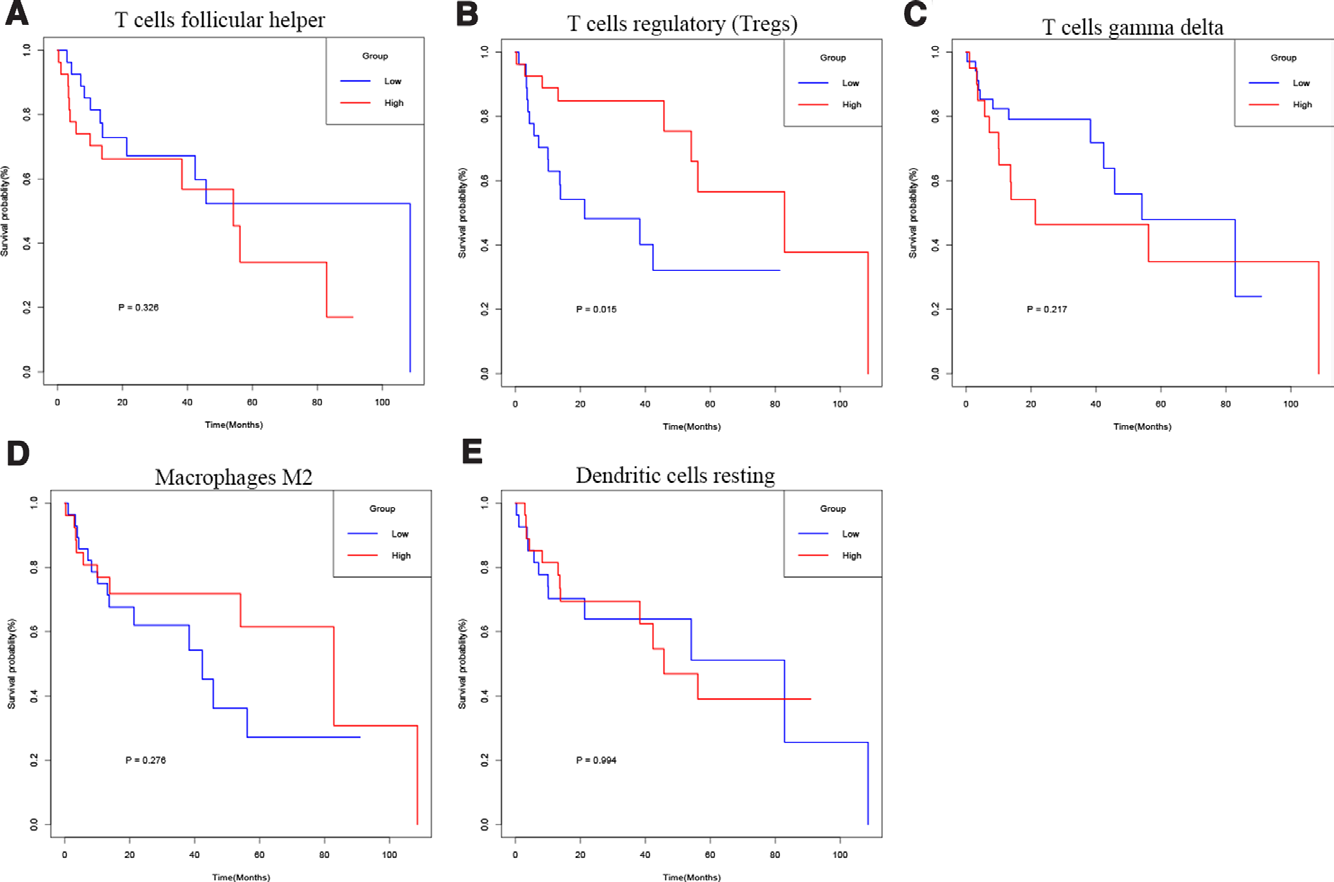
Survival analysis of immune cells with significant differences.

**Figure 4.**
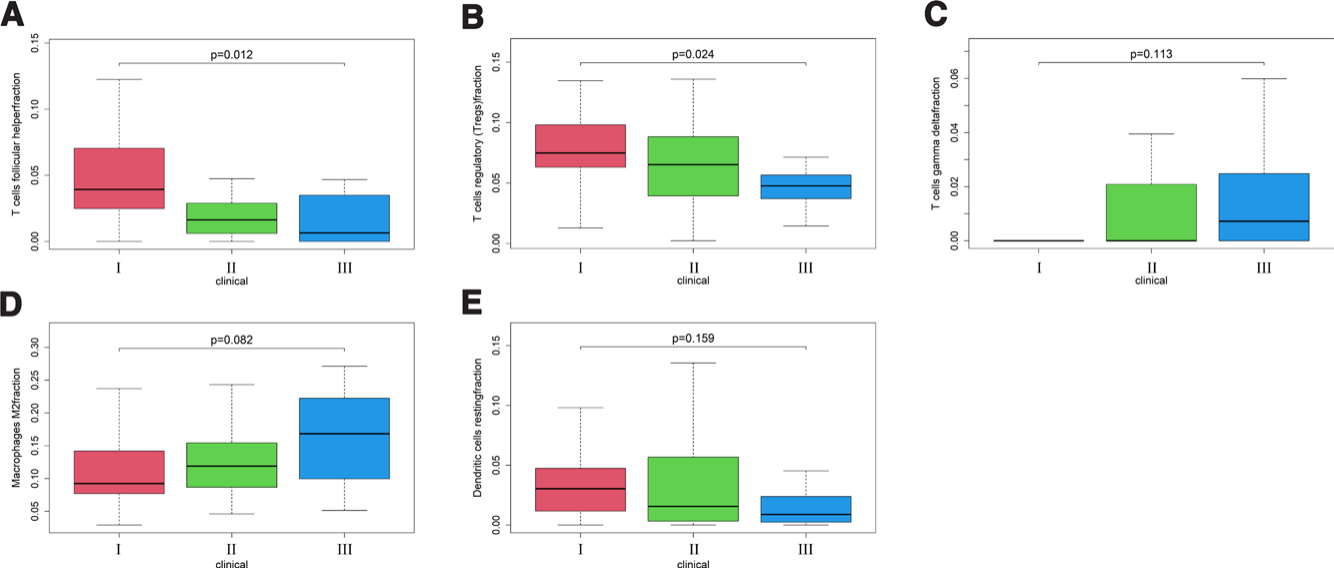
Clinical stage difference of immune cells with significant differences.

On this basis, I conducted enrichment Heatmap analysis (Fig. 1B) and correlation analysis (Fig. 1C) on immune cells, and compared them according to the stage of patients, especially stage I and stage III (Fig. 2C). I found that there are several immune cells with high differences: T cells follicular helper, T cells regulatory (Tregs), T cells gamma delta, Macrophages M2, Dendritic cells resting. Therefore, I analyzed the survival of patients based on the expression of significantly different immune cells (Fig. 3). T cells follicular helper (Fig. 3A) and Tregs (Fig. 3B) have obvious differences in different stages of HCC. Then, I extracted the expression of these significantly different immune cells in stage I, stage II, and stage III, and analyzed their data to obtain these results (Fig. 4). It can be seen from the results that there are significant differences in the prognosis between Tregs and HCC stage, and the decrease of Tregs content from stage I to stage III is significant (Fig. 4B), and the impact on survival and prognosis is also significantly different. According to the above situation, I believe that Tregs has a more obvious effect on HCC. High content of Tregs is harmful to the survival and prognosis of HCC, and low content of Tregs is beneficial to the survival and prognosis.

### 3.3. Screening and determination of differential genes

Therefore, I began to look for differential genes, that is, differential genes that can regulate Tregs, and differential genes have a significant impact on the survival and prognosis of patients with HCC. The screening of differential genes is based on the critical value set by log2 fold change >1 and Padj <0.05. After screening differential genes, I used the survival data in TCGA database (n = 463) for Cox regression analysis, and established a Cox regression analysis model to screen the top gene, in which *P*-value <.05 and the 95% confidence interval of gene risk ratio does not include 1. Because the high expression of Tregs has a bad effect on the survival and prognosis of HCC, the genes I selected are divided into 2 types. One is the gene of risk factor. When such gene is highly expressed, the expression of Tregs should be increased; When the expression of these genes is low, the expression of Tregs should be reduced, that is, these genes should be positively correlated with Tregs. The other is genes with protective factors. When such genes are highly expressed, Tregs should be lowly expressed; When the expression of such genes is low, the expression of Tregs should be increased, that is, such genes should be negatively correlated with Tregs. So, I found the gene CENPO.

### 3.4. Analysis of differential genes

**Figure 5:**
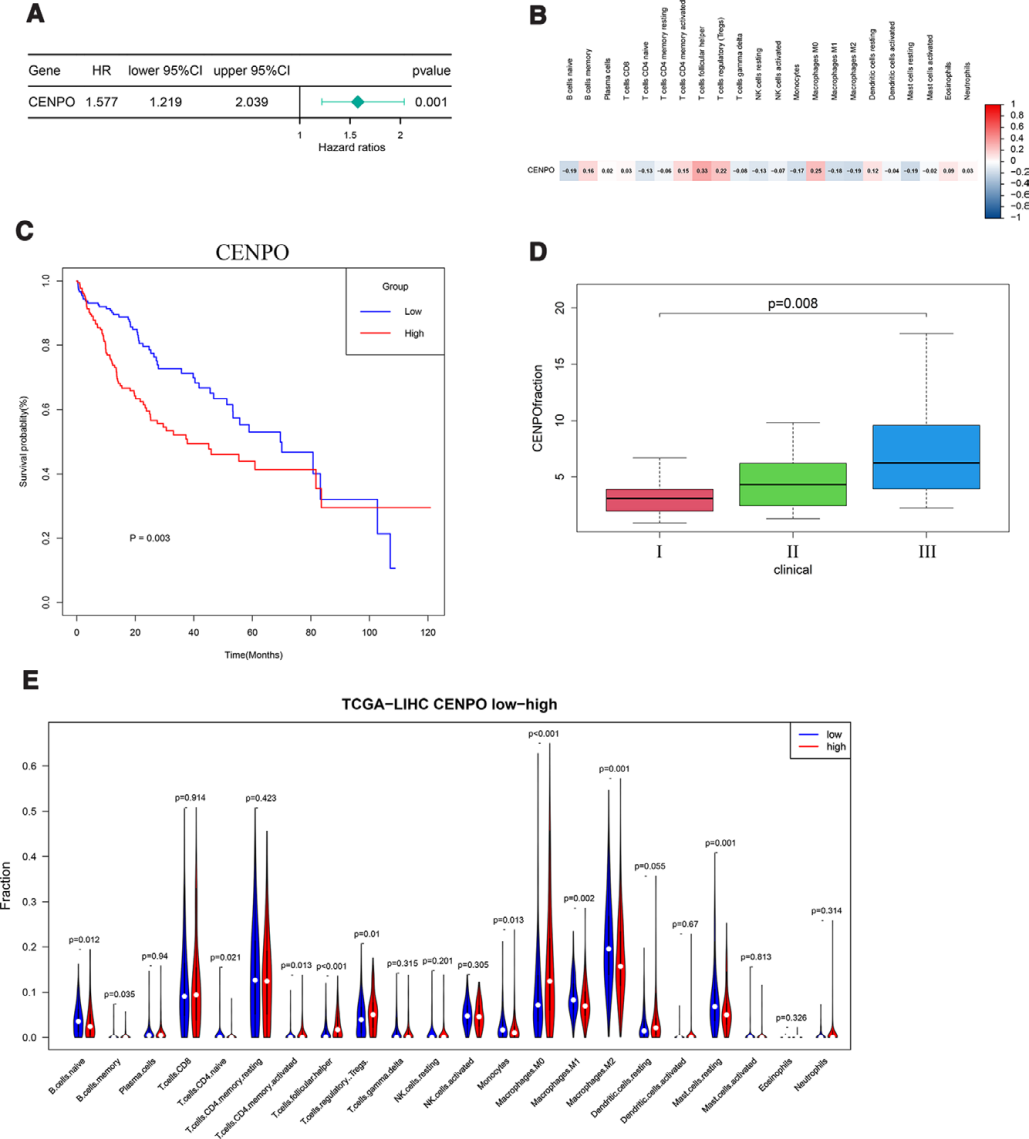
(A) Forest map of CENPO. (B) The correlation between CENPO and 22 immune cells was analyzed by Spearman. (C) Survival analysis of CENPO in TCGA database. (D) Clinical stage difference of CENPO. (E) According to the median of CENPO, it is divided into low expression and high expression, and the difference between immune cells and their expression is calculated by Wilcoxon rank sum. CENPO = centromere protein o, TCGA = The Cancer Gene Atlas.

**Figure 6:**
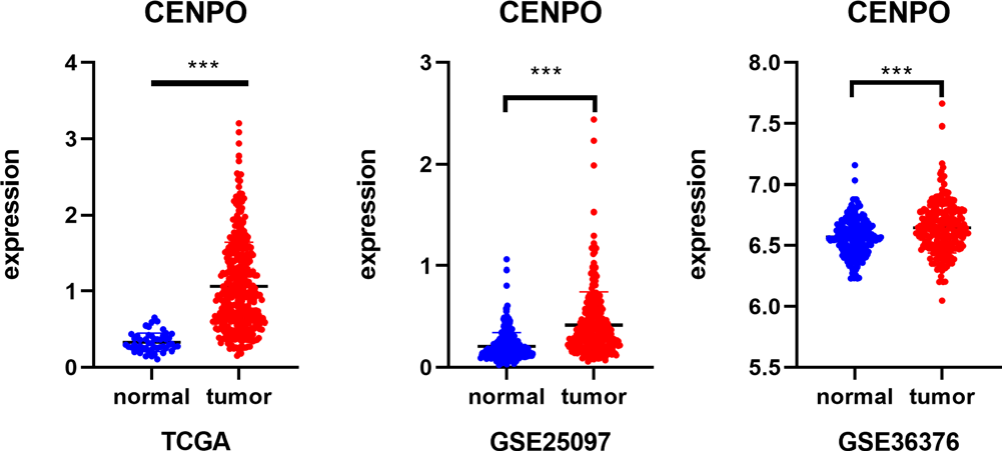
(A) The difference of CENPO expression between normal tissue and tumor tissue in TCGA database. *P < .05, **P < .01, ***P < .001. (B) The difference of CENPO expression between normal tissue and tumor tissue in GSE25097. *P < .05, **P < .01, ***P < .001. (C) The difference of CENPO expression between normal tissue and tumor tissue in GSE36376. *P < .05, **P < .01, ***P < .001. CENPO = centromere protein o, TCGA = The Cancer Gene Atlas.

According to Cox regression analysis, gene CENPO is a gene with risk factors, so I drew the forest map according to the results of Cox regression analysis (Fig. 5A). According to the above inference, CENPO should be negatively correlated with the expression of Tregs (Fig. 5B). In addition, I need to know whether CENPO have significant differences in the survival and prognosis of HCC. I analyzed their survival data and drew their survival curve (Fig. 5C). In addition to the above studies, the expression content of CENPO in stage is also different, and there is an upward trend from stage I to stage III (Fig. 5D). Therefore, I calculated and plotted the correlation between CENPO and Tregs, whether the expression of the gene is related to the expression of 22 immune cells, and especially the content of Tregs (Fig. 5E). The results show that there was a significant difference between CENPO and the content of Tregs, which was negatively correlated with Tregs, and there was also a significant difference in the survival and prognosis of HCC. I used 2 GEO datasets GSE25097 and GSE36376 to verify whether CENPO is significantly different from adjacent HCC (Fig. 6).

### 3.5. Gene ontology and KEGG enrichment analysis

**Figure 7:**
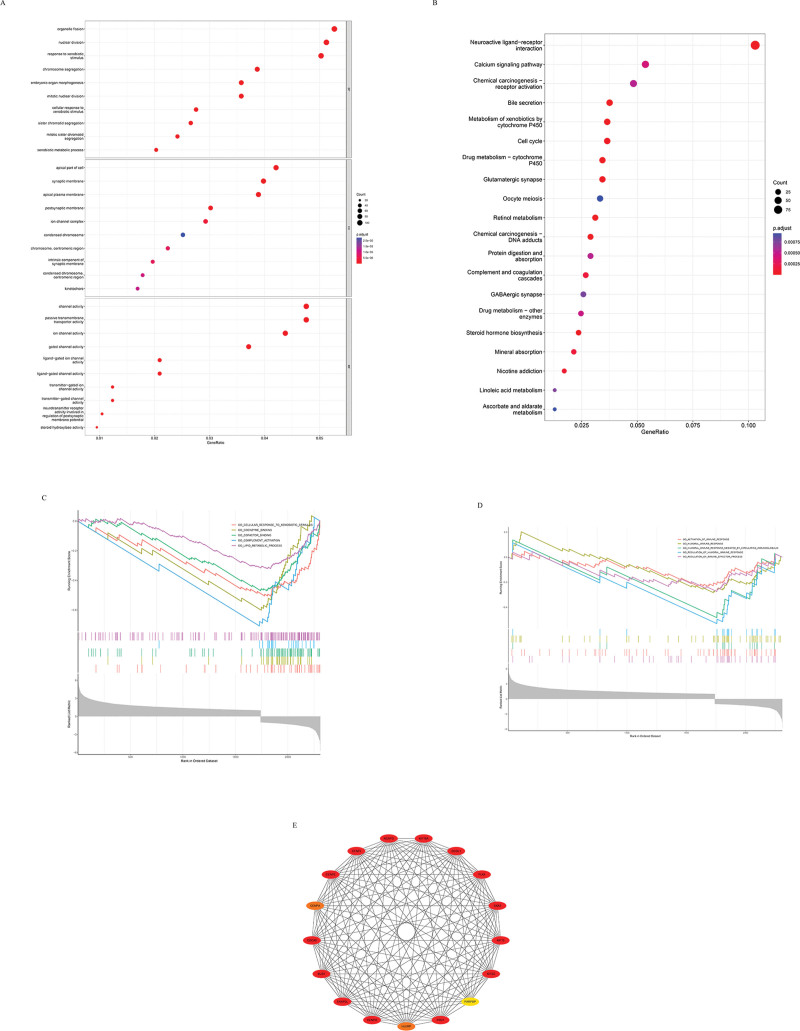
(A) Go enrichment analysis of CENPO. (B) KEGG enrichment analysis of CENPO. (C) The first 5 enrichment pathways of CENPO’s GSEA. (D) Immune related enrichment pathways of GSEA of CENPO. (E) PPI network diagram after CENPO co expression analysis. CENPO = centromere protein o, GSEA = gene set enrichment analysis, KEGG = kyoto encyclopedia of genes and genomes, PPI, protein protein interaction.

In order to better understand the expression of these genes in HCC, I conducted go and KEGG enrichment analysis to analyze the aggregation of CENPO for metabolic pathways and pathways of HCC. According to go analysis, CENPO is mainly enriched in organelle fission, nuclear division and response to xenobiotic stimulus in molecular function, in apical part of cell, synaptic membrane and apical plasma membrane in cellular component, and in channel activity and passive transporter activity in biological process (Fig. 7A). According to KEGG analysis, the pathway enrichment of CENPO on HCC is mainly in neuroactive live receptor interaction (Fig. 7B).

### 3.6. Gene set enrichment analysis

In order to explore the enrichment of up-regulated and down-regulated pathways of this gene, I performed GSEA, and I extracted the first 5 and immune related pathways. The results show that CENPO down regulates these pathways (Fig. 7C and D).

### 3.7. PPI network

Firstly, I use CIBOPORTAL database (http://www.cbioportal.org/)^[25]^ to analyze the co-expression genes of CENPO and screen out the genes with more than 0.7 correlation with CENPO. Then I use the string tool to establish the PPI network of these genes to analyze the relationship between CENPO and other genes, select the genes known to interact with CENPO and make the PPI network (Fig. 7E). The redder the color, the higher the degree.

## 4. Discussion

In our study, our main purpose is to find the difference of immune cell infiltration in different stages of HCC and screen out the differential genes that may regulate highly differential immune cells, so as to improve the survival and prognosis of patients with HCC. TME has a very key and important impact on the occurrence and development of tumor, survival and prognosis of patients. Therefore, I need to explore the potential therapeutic targets of tumor in TME, change TME and immune infiltration, and inhibit tumor development.^[4,26]^ A large number of studies have shown that immune microenvironment plays an important role in tumors. Many tumor immune mechanisms have been explored and many immune targets have been found as treatment schemes.^[27]^ I analyzed the RNA SEQ data of LIHC data in TCGA database. The results show that immune cells in TME had an important impact on the survival and prognosis of HCC. In recent years, targeted therapy has gradually been applied to the clinical treatment of HCC. In recent years, targeted therapy has gradually been applied to the clinical treatment of HCC. The application of regorafenib and sorafenib indicates the full opening of the second-line treatment of HCC, and more effective new drugs and the most influential immune checkpoint inhibitors (ICIs) have emerged, which have made a great contribution to prolonging the survival of patients with HCC.^[28]^ Regulatory T cells (Tregs) is a subgroup of CD4 + T cells, which plays an indispensable role in immune tolerance.^[29]^ Tregs also play a very important role in the TME. The immunosuppressive activity of Tregs is one of the mechanisms to promote tumor development.^[30]^ Tregs in TME not only reduce the efficiency of anti-tumor immune response, but also support the immune escape mechanism of tumor cells and promote the proliferation and development of tumor, so Tregs has become one of the targets of immunotherapy.^[31]^ There is a complex relationship between Tregs and ICIS. The depletion and high content of Tregs make the survival rate of patients low and the prognosis poor. ICIS can induce effector T cells to produce antitumor effect. However, Tregs mediated ICIS is easy to obtain drug resistance, which makes us need to further improve the treatment scheme, reduce or even overcome the drug resistance mechanism, and find new immunosuppressive points.^[31,32]^

Therefore, I screened the gene CENPO, also known as mcm21r, icen-36. However, the function of CENPO is not clear.^[33]^ According to the introduction of National Center for Biotechnology Information (https://www.ncbi.nlm.nih.gov/), this gene is necessary in mitosis and encodes alternative splicing transcriptional variants of many protein subtypes. It is reported that CENPO can promote the proliferation of gastric cancer and has a negative impact on the prognosis of gastric cancer.^[34]^ Studies have also shown that upregulation of CENPO may lead to bladder cancer and may depend on P53 to regulate the development, proliferation and apoptosis of colorectal cancer.^[35,36]^ Studies have suggested that the high expression of CENPO has an adverse effect on the survival of breast cancer and leads to poor prognosis. It is also considered that CENPO is an independent factor affecting the distant recurrence survival rate of breast cancer patients.^[37]^ The role of this gene CENPO is gradually being developed and explored by researchers, and seems to be related to mental diseases.^[38–40]^ In this study, I found that CENPO is also closely related to the prognosis of HCC. CENPO has a positive correlation with Tregs in the TME of HCC, and high expression of CENPO increases the content of Tregs, which can be consumed to make the tumor produce anti immunity or immune escape, and promote the development of HCC. Moreover, the survival rate of HCC with high expression of CENPO is low and the prognosis is poor.

The data in this study only come from TCGA database and GEO database, and the data analysis only exists at the theoretical level, which cannot be verified in animal model or cell experiment. I hope that the verification can be completed in the future.

## 5. Conclusion

In conclusion, this is a preliminary study on the survival and prognosis of HCC based on TCGA database and GEO database. In this study, I searched and analyzed the effect of immune microenvironment on HCC, and found CENPO, which may be a potential target for the treatment of HCC. I hope that on this basis, this study can be experimentally verified in the future. Whether CENPO has an impact on the prognosis of HCC and whether it has an effective effect on the prognosis of human HCC. I sincerely hope that my research can provide new therapeutic targets and new directions and ideas for the treatment of HCC.

## Author contributions

**Conceptualization:** Zhi-Wei Xu.

Data curation: Zhi-Wei Xu.

Formal analysis: Zhi-Wei Xu.

Methodology: Zhi-Wei Xu.

Writing – original draft: Zhi-Wei Xu.

Writing – review & editing: Zhi-Wei Xu.
